# Shortening the Time Interval for the Referral of Patients With Soft Tissue Sarcoma to Expert Centers Using Mobile Health: Retrospective Study

**DOI:** 10.2196/40718

**Published:** 2022-11-09

**Authors:** Simon Nannini, Nicolas Penel, Emmanuelle Bompas, Thibault Willaume, Jean-Emmanuel Kurtz, Justine Gantzer

**Affiliations:** 1 Department of Medical Oncology Strasbourg-Europe Cancer Institute Strasbourg France; 2 Department of Medical Oncology Center Oscar Lambret Lille University Lille France; 3 Department of Medical Oncology Institut de Cancérologie de l’Ouest Nancy France; 4 Department of Radiology University Hospital of Strasbourg Strasbourg France

**Keywords:** sarcoma, apps, mHealth, mobile health, health app, mobile app, referral, consultation, care coordination, tumor, cancer, oncology, soft tissue, connective tissue, prognosis, communication, interprofessional, patient management, physician, doctor, health care provider, specialist, general practitioner, GP

## Abstract

**Background:**

According to guidelines, all patients with sarcoma must be managed from initial diagnosis at expert sarcoma centers. However, in everyday practice, the time interval to an expert center visit can be long, which delays presentation to an expert multidisciplinary tumor board and increases the risk of inappropriate management, negatively affecting local tumor control and prognosis. The advent of mobile health offers an easy way to facilitate communication and cooperation between general health care providers (eg, general practitioners and radiologists) and sarcomas experts. We developed a mobile app (Sar’Connect) based on the algorithm designed by radiologists from the French Sarcoma Group. Through a small number of easy-to-answer questions, Sar’Connect provides personalized advice for the management of patients and contact information for the closest expert center.

**Objective:**

This retrospective study is the first to assess this mobile app’s potential benefits in reducing the time interval for patient referral to an expert center according to the initial clinical characteristics of the soft tissue tumor.

**Methods:**

From May to December 2021, we extracted tumor mass data for 78 patients discussed by the multidisciplinary tumor boards at 3 centers of the French Sarcoma Group. We applied the Sar’Connect algorithm to these data and estimated the time interval between the first medical description of the soft tissue mass and the referral to expert center. We then compared this estimated time interval with the observed time interval.

**Results:**

We found that the use of Sar’Connect could potentially shorten the time interval to an expert center by approximately 7.5 months (*P*<.001). Moreover, for half (31/60, 52%) of the patients with a malignant soft tissue tumor, Sar’Connect could have avoided inappropriate management outside of the reference center. We did not identify a significant determinant for shortening the time interval for referral.

**Conclusions:**

Overall, promoting the use of a simple mobile app is an innovative and straightforward means to potentially accelerate both the referral and management of patients with soft tissue sarcoma at expert centers.

## Introduction

Sarcomas are malignant mesenchymal tumors that can arise from any soft tissue or bone. Soft tissue sarcoma (STS) represents less than 1% of adult cancers and is characterized by important heterogeneity in clinical presentation, histological subtypes, and aggressiveness [1]. In general, the diagnosis and management of sarcomas and soft tissue intermediate malignancies can be inappropriate at nonexpert centers [2]. In France, only 40% to 50% of patients with sarcoma are treated according to clinical guidelines [3], even though following these guidelines is an independent prognostic factor for both disease-free and overall survival [4]. Nevertheless, a considerable proportion of patients with sarcoma, particularly those with smaller soft tissue tumors (STTs), undergo inappropriate surgery (eg, “whoops” surgery) [5-7] outside of an expert center, sometimes without any prior imaging being conducted. Such initial inappropriate surgical procedures lead to the worst local tumor control and poor survival [8,9]. Blay et al [10] report that surgery at an expert center significatively improves overall survival with a hazard ratio of 0.68 compared to surgery outside an expert center. Furthermore, other treatments may become necessary after R1/R2 resection, such as re-excision, with the risk of higher rates of mutilating surgical procedures or the use of adjuvant radiotherapy or chemotherapy leading to more side effects [11-13]. Despite the addition of these treatments, previous studies have shown that unplanned surgery is always associated with poor outcomes [14,15].

The reasons for “whoops” surgery include difficulty in clinically discriminating benign from malignant soft tissue masses [16]. Indeed, sarcomas can mimic lipoma, benign neurogenic tumor, certain infections, and thrombosis, among others [17-21]. Regardless, certain criteria suggestive of STS, including a size over 5 cm, tumor depth, recent growth, and pain, should lead to magnetic resonance imaging (MRI) assessment [20,22,23]. A suspicious tumor can present an inhomogeneous density distribution due to complex pathological changes, including bleeding, necrosis, walls, and calcification, as opposed to the usual simple structure and more homogenous density distribution of a benign STT [24]. As such, the French Sarcoma Group (FSG) expert radiologist team developed an algorithm to guide radiologists in identifying benign versus nonbenign STTs based on the first imaging results [25].

Another reason that could explain this disparity in the management of patients with sarcoma is the social and geographical factors of an area that can substantially delay reach to expert centers, especially for mountainous and precarious districts [4]. However, the development of the FSG across the country helped to reduce the risk of nonoptimal management by proposing an app to help identify and contact the closest expert center—thus promoting a more homogeneous management for patients with sarcoma [25].

Shortening the time interval for referral to an expert center for patients with STS is challenging. To reach most health care providers and raise their awareness, we developed, with the help of Chlorophyll Vision, a mobile app called Sar’Connect in 2021, which aims to increase the rate of early detection of STS and facilitate patient referral to expert centers based on the FSG radiological algorithm and geolocation of health professionals [26]. The FSG radiological algorithm was adapted into a list of 3 to 6 short-answer questions based on clinical and radiologic anonymized information. After answering these questions, users receive 1 of the 3 available pieces of advice for orientation: direct referral to an expert center, necessary complementary imaging, or possible nonexpert center management (Figure S1 in Multimedia Appendix 1). To our knowledge, this is the first time that this kind of app was developed for the management of sarcoma. This study is also the first to assess the potential benefits of Sar’Connect, which has been available since April 2021, for the referral and management of patients with STTs [26].

## Methods

### Study Design and Recruitment

In this retrospective in silico study, data were collected from 3 different FSG expert centers from May to December 2021: “Centre Oscar Lambret” (Lille), “Institut de Cancérologie de l’Ouest” (Nantes), and “Institut de Cancérologie de Strasbourg Europe” (Strasbourg).

### Ethics Approval

Patient data reported in a multidisciplinary tumoral board (MTB) from an FSG expert center were saved in the NetSarc+ database, which centrally reviewed them. Patients were warned of this process before discussion in MTB and were free to refuse the use of their data. The NetSarc+ data collection and further analysis were approved by the ethics committees as required by the applicable national legislation: approval by the “Comité consultatif sur le traitement de l’information en matière de recherche dans le domaine de la santé” on September 16, 2010 (authorization number 10.403), and approval by the “Comité National Informatique et Liberté” on the July 15, 2013 (authorization number 910390, Decision DR-2013-383). No information used in the study or the app was saved.

### Patients

Using a structured questionnaire, we collected data for patients with soft tissue masses discussed by an MTB from an FSG expert center. Patient eligibility was as follows: (1) all consecutive cases of histopathological diagnosis confirmed by expert pathologist from “Réseau de Référence en Pathologie des Sarcomes” and (2) cases discussed at the time of initial diagnosis. We excluded patients with primary bone tumors and those with carcinoma, melanoma, or hematological malignancies. In the case of Ewing sarcoma, osteosarcoma, or chondrosarcoma, patients were only included if the tumor was extraskeletal and diagnosis was performed based on the biopsy of a STT. The data collected included histology; date and medical decisions following the first description of the STT; date and conclusion from the first MTB; and tumor characteristics (largest diameter, localization, and, if available, the type and conclusions of radiological exams). We defined 2 groups: benign tumors and malignant tumors (including sarcomas and intermediate tumors). Statistical analyses were performed for each group, both combined and separately.

### Assessments

Calculation of the interval was based on the following 2 dates: (1) the date of the first medical description of the soft tissue mass, as mentioned in the medical files; and (2) the date of the first consultation or discussion in the MTB at the expert center.

The mobile app was used retrospectively with the medical information available during the first medical description of the STT. To run the app, the data needed are the size of the mass in millimeters; its depth (superficial or deep); its radiological features (lipid composition, homogeneity, or heterogeneity); and its clinical characteristics (growth, pain, hardness, and shrinkage of the mass). No data are stored, and all data are completely anonymous to protect medical privacy. According to this information, 3 outcomes are possible: (1) advice for direct referral to an expert center, (2) advice to undergo a complementary radiological exam (especially MRI), and (3) informed of the possibility to manage this mass at a local center. To match these 3 possible outcomes of Sar’Connect, an estimated time interval before referral to an MTB was associated with each of them, as follows. To simulate an acceptable and conservative time interval for each outcome, we assigned a 1-month interval for direct referral to an expert center and a 2-month interval if a complementary radiological exam was advised by the algorithm prior to referral. When the algorithm suggested possible nonexpert center management, an interval of 0 months was assigned in the case of benign tumors and an interval equal to the real-life observed time to referral was assigned in the case of nonbenign tumors. All estimated time intervals were assigned according to local practice.

### Study Objectives

The primary objective was to compare the real-life observed time to referral to an expert center (calculated between the first medical description of the STT and the date of the MTB) with the time estimated retrospectively according to the recommendations of the algorithm. Secondary objectives included identifying the determinants of the time interval. We also assessed the potential rate of benign tumors being referred to an expert center after the use of Sar’Connect.

### Statistical Analysis

To obtain complete data for at least 40 patients, a sample of 60 patients was required for this study. This sample size was estimated to provide at least 90% power for an expected difference of 8 months in favor of Sar’Connect. This difference value and sample size were chosen according to a previous exploratory unpublished retrospective analysis conducted with the same methodology as the one described in this study and based on records from the MTB of Strasbourg’s expert center. According to our local practice, this delay was substantial for this situation. Patient characteristics are described using numbers and proportions for categorical variables and using the mean, SD, median, and IQR for continuous variables.

We compared the actual observed time with the estimated time to an expert center using 2-tailed Student *t* test for matching. Sensitivity analysis was conducted based on the change in the initial time interval from 1 to 3 months in cases of immediate referral to an expert center and from 2 to 6 months when an additional imaging procedure was needed. The results are presented as differential means with their CIs and *P* values. All tests were performed with a 2-sided α risk of 5%. Statistical analyses were performed with R statistical software (version 4.1.2; R Foundation for Statistical Computing).

To identify the determinants of the time interval to referral, a Pearson test was performed on the following putative determinants: distance between the patient’s home address and the nearest expert center, age, number of clinical signs, tumor size and depth, and prior imaging procedure. All tests were performed first on the whole cohort and then on patients with malignant tumors.

For exploratory analyses, a Bayesian-based method was performed to describe the predictive impact of selected variables using Markov chain Monte Carlo methods. Statistical significance was established when the 95% credibility interval did not contain 0. The results are presented as the median and 95% credibility interval.

## Results

### Patient Inclusion

**Figure 1:**
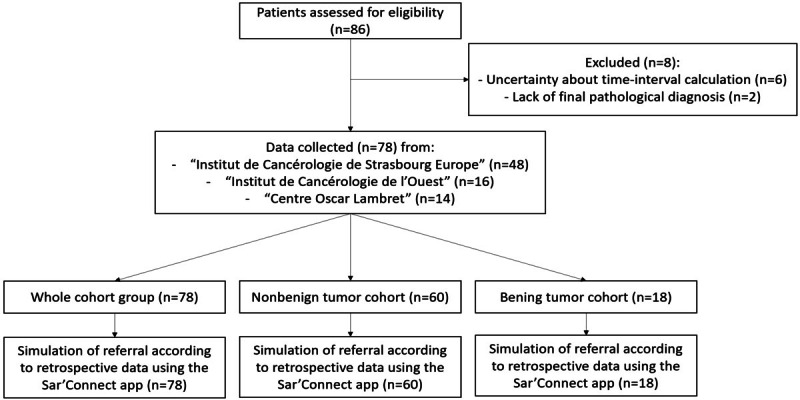
Flowchart of patient enrollment.

In total, 86 patients were included from May to December 2021 (Figure 1). Among them, 8 were excluded from the study: 2 because of the lack of a final pathological diagnosis and 6 because of uncertainty about the time-interval calculation. For the remaining 78 patients, 18 had a benign tumor and 60 a malignant tumor. Main data are summed in Figure S2 in Multimedia Appendix 1.

### Patients With Nonbenign STT

#### General Patient Characteristics

We collected data for 60 patients diagnosed with sarcoma or a locally malignant tumor (Table 1). Of the 60 patients in the cohort, 33 (55%) were women. The median age was 60.5 (IQR 21-92) years, with only 6 (10%) aged >30 years. According to medical histories, 10 (17%) patients had a history of 1 or more cancers, including 3 cases of prostate cancer, 2 cases of breast cancer, 3 cases of skin basal cell carcinoma, 2 cases of colorectal cancer, 1 case of endometrial cancer, and 1 case of T-cell lymphoma. There was 1 (2%) patient who had a genetic predisposition toward sarcoma due to neurofibromatosis 1.

The median distance between the patient’s hometown and the nearest expert center was 69 (IQR 1-1103) km. Only 1 patient was not referred to the nearest expert center owing to personal choice.

The histological subtype distribution was as expected. Of the 60 patients, 15 (25%) were diagnosed with liposarcoma, including 7 (12%) cases of atypical lipomatous tumors/well-differentiated liposarcoma; 9 (15%) had leiomyosarcoma; 6 (10%) had undifferentiated pleomorphic sarcoma; and 6 (5%) had desmoid-type fibromatosis. Additionally, 15 (25%) cases were grouped as “other histological subtypes” (including extraskeletal Ewing sarcoma, extraskeletal osteosarcoma, gastrointestinal stromal tumor, perivascular epithelioid cell tumor, giant cell tumor, synovial sarcoma, angiosarcoma, and clear-cell sarcoma).

The median tumor size was 90 (IQR 10-450) mm; 10 tumors were not palpable. Regarding location, 26 (43%) tumors were in the limbs, and 25 (42%) were in the trunk (including the pelvis, mediastinum, retroperitoneum, and paratesticular spermatic cord); 9 (15%) patients had a tumor of the thoracic or abdominal wall. Initial clinical signs reported by patients were tumor growth (n=40, 67%), pain (n=25, 42%), and STT stiffness (n=9, 15%).

Radiological exam data showed a median tumor size of 102 (IQR 11-320) mm. Out of 57 tumors, tumor size was over 50 mm in 44 (77%) patients and less than 30 mm in only 4 (7%). Out of 54 tumors, only 10 (19%) cases were superficial and 44 (81%) were at least partially deep. Of the 60 patients, 11 (18%) did not undergo any imaging prior to MTB presentation.

**Table 1:** Patient and tumor characteristics of the nonbenign tumor cohort (N=60).

Population characteristic		Value
**Age (years)**		
	Median (IQR)	60.5 (21-92)
	<30, n (%)	6 (10)
	30-60, n (%)	24 (40)
	≥60, n (%)	30 (50)
**Sex**		
	Female	33 (55)
	Male	27 (45)
**Geographical data**		
	Distance between the patient and nearest expert center (km), median (IQR)	69 (1-1103)
**Medical history, n (%)**		
	Other cancers^a^	10 (17)
	Familial history of cancer	3 (5)
	Genetic predisposition (neurofibromatosis 1)	1 (2)
**Histology^b^, n (%)**		
	Liposarcoma	15 (25)
	Leiomyosarcoma	9 (15)
	Undifferentiated pleomorphic sarcoma	6 (10)
	Myxofibrosarcoma	4 (7)
	Desmoid tumor	3 (5)
	Dermatofibrosarcoma	2 (3)
	Solitary fibrous tumor	2 (3)
	Malignant peripheral nerve sheet tumor	2 (3)
	Rhabdomyosarcoma	2 (3)
	Other	15 (25)
**Tumor size (mm; N=57)^c^**		
	Clinical evaluation, median (IQR)	90 (10-450)
	Radiological evaluation, median (IQR)	102 (11-320)
	<30, n (%)	4 (7)
	30-50, n (%)	9 (16)
	≥50, n (%)	44 (77)
**Clinical manifestation^d^, n (%)**		
	Pain	25 (42)
	Progression of the tumor	40 (67)
	Hardness	9 (15)
	Shrinkage of the tumor	1 (2)
	Recurrence	0 (0)
**Location^e^, n (%)**		
	Limb	26 (43)
	Trunk	25 (42)
	Abdominal or thoracic wall	9 (15)
**Depth on radiological exam (N=54)^f^, n (%)**		
	Superficial tumor	10 (19)
	Deep tumor or both superficial and deep	44 (81)

^a^Patients could present more than 1 type of other cancer; other cancers included 3 patients with prostate cancer, 2 patients with breast cancer, 3 patients with epidermal carcinoma of the skin, 2 patients with colorectal cancer, 2 patients with endometrial cancer, and 1 patient with T-cell lymphoma.

^b^Other histological subtypes include other soft tissue sarcomas, gastrointestinal stromal tumors, and extraskeletal Ewing sarcoma or osteosarcoma.

^c^Missing data (n=3).

^d^Patients could have more than 1 symptom.

^e^Trunk localization included the peritoneum, mediastinum, retroperitoneum, and paratesticular spermatic cord.

^f^Including 10 nonpalpable soft tissue tumors; missing data (n=6).

#### Impact on Patient Referral and Appropriate Care

We assessed the decision for patient management according to the first descriptive data (Table 2).

Of the 60 patients, 27 (45%) were referred to an expert center for initial management, including 18 (30%) who underwent imaging before their referral. For 34 (92%) out of 37 patients, imaging diagnosed an atypical or suspicious aspect of the STT compatible with sarcoma. Despite these conclusions, half (17/34, 50%) of the cases were managed at a nonexpert center.

Out of 60 cases, 22 (37%) were managed at a nonexpert center for biopsy (n=9, 15%) or surgery (n=13, 22%) after the discovery of the STT. Follow-up was the only decision for 11 (18%) cases.

Retrospective simulated outcomes for referral from using the Sar’Connect app with the initial data would have recommended a direct referral to the nearest expert center for 46 (77%) patients and complementary imaging (ultrasound echography or MRI) for 13 (22%) patients. The mobile app algorithm suggested a major change in care for 31 (52%) patients versus real-life outcomes; 30 (50%) patients would have an estimated adequate referral with the use of Sar’Connect, whereas 1 (2%) patient with a superficial dermatofibrosarcoma less than 1 cm in size would not have been referred to an expert center.

**Table 2:** Patient follow-up and management in the nonbenign tumor cohort (N=60).

Details on the first management of soft tissue tumors		n (%)
**Radiological exam^a^**		
	Ultrasound echography	22 (37)
	MRI^b^	34 (57)
	Computed tomography	24 (40)
	None	11 (18)
**Results of radiological exams^c^ (N=37)**		
	Atypical or suspicious aspect of soft tissue tumor	34 (92)
	Homogeneous adipose or typical aspect of “pseudotumor”	3 (8)
**Real-life management after discovery of the mass**		
	Follow-up without radiological exams	5 (8)
	Follow-up with periodic radiological exams	6 (10)
	Biopsy at a nonexpert center	9 (15)
	Surgery outside of an expert center	13 (22)
	Referral to an expert center	27 (45)
**Simulated outcomes of** **Sar’Connect** **according to first encounter data**		
	Referral to an expert center	46 (77)
	Imaging (MRI or echography)	13 (22)
	Referral to a nonexpert center	1 (2)
**Potential difference of management between real-life observed outcome and Sar’Connect advice^d^**		
	Difference	31 (52)
	No difference	29 (48)

^a^Patients could have more than one type of radiological exam.

^b^MRI: magnetic resonance imaging.

^c^37 patients with radiologist conclusion available (11 patients without radiological exam); the suspicious aspect of the soft tissue tumor could contain heterogeneity tissue, anarchic vasculature, enhancement, or thick wall. Pseudotumor included synovial or rheumatism degeneration, vascular or lymphatic malformation, elastofibroma, Morton neuroma, hemangioma, schwannoma, and glomus tumor.

^d^Difference in decision was based on groups: optimal decision (a sarcoma referred to expert center) and nonoptimal decision (nonbenign soft tissue tumor referred to a nonexpert center or under watchful waiting only).

### Patients Diagnosed With Benign Tumors

Of the 60 patients, 18 (30%) were diagnosed with benign tumors. Their characteristics are reported in Table S1 in Multimedia Appendix 1.

Of the 18 patients, 11 (61%) were referred to an expert center, and all (n=18, 100%) had prior imaging. MRI was performed for 14 (78%) patients, with an atypical or suspicious STT described in most (n=11, 61%) cases. Additionally, 3 (6%) patients were not initially referred to an MTB after imaging, and 1 (6%) patient underwent surgery at a nonexpert center before being referred to an MTB. Using Sar’Connect, 13 (72%) patients would have been directly referred to the expert center, and only 2 (11%) cases would have been recommended for possible management at a nonexpert center. Overall, we found a potential difference in management between the real-life outcome and Sar’Connect simulation in 9 (50%) cases. The mobile app suggested more appropriate management for 4 (22%) patients and referred 5 (28%) with benign tumors to an MTB (Table S2 in Multimedia Appendix 1).

The benign tumor group was not large enough to allow for comparisons with the nonbenign STT group. Nevertheless, variables that appeared to be numerically different included a higher median clinical and radiological size of the tumor (60 and 40 mm in the benign group versus 90 and 120 mm in the nonbenign STT group, respectively) and the absence of a painful STT in all (18/18, 100%) patients with benign tumors compared to 25 (42%) out of 60 patients in the nonbenign population.

### Impact on the Time Interval to Appropriate Management

Analyses were conducted on the whole cohort (78 patients), including patients with benign and nonbenign STTs. With a mean time interval of 9.14 (IQR 1-85) months in real life versus 1.4 (IQR 0-10) months estimated with Sar’Connect, we found a potentially clinically meaningful reduction in this time interval of 7.7 months (*P*<.001). To validate this difference, we repeated the analysis with a less optimistic estimated time interval for each Sar’Connect result. Hence, we assigned a 3-month time interval in cases of advice for direct referral and a 6-month time interval in cases of radiological exam requirement. This sensitivity analysis found a mean application time interval of 3.6 months and a potential estimated reduction of 5.4 months in favor of the mobile device algorithm (*P*=.002; Table 3). Two values were considered extreme data (designed as time higher than 2.5 times the SD), and statistical analysis excluding these data confirmed a simulated 6-month difference (*P*<.001).

We repeated the same analyses in the nonbenign STT population (Table 4) and, again, retrospectively using Sar’Connect to guide patient referral, which resulted in an estimated potential benefit of 6.5 months (*P*<.001). Although the difference was smaller, real-life outcomes occurred in a significant number of 13 (22%) patients who underwent surgery (with or without prior imaging) or biopsy (n=9) at a nonexpert center.

**Table 3:** Time interval before patient referral to an expert center in the whole cohort (N=78).

Time interval for patient referral to an expert center		Months	*P* value
**Real-life observed results, mean (IQR)**			
	Time interval after radiological exam	9.14 (1-85)	
	Time interval after biopsy of the tumor at a nonexpert center	8.11 (2-36)	
	Time interval after surgery outside of an expert center	7.18 (2-28)	
	Time interval for the whole cohort	9.0 (1-85)	
**According to simulation by** **Sar’Connect**			
	Time interval, mean (IQR)	1.4 (0-10)	
	Difference with real-life observed results, mean (95% CI)^a^	7.7069 (4.3677-11.046)	<.001
**Sensitivity test following Sar’Connect^b^**			
	Time interval, mean (IQR)	3.63 (0-10)	
	Difference with real-life observed results, mean (95% CI)^a^	5.37 (2.0293-8.7178)	.002

^a^Mean difference calculated with 2-tailed Student *t* test for matched data.

^b^Results of the mobile app in sensitivity analysis by replacing the 1-month time interval with a 3-month interval and the 2-month time interval with a 6-month interval.

**Table 4:** Time interval before patient referral to an expert center in the nonbenign tumor cohort (N=60).

Time interval for patient referral to an expert center		Months	*P* value
**Real-life observed results, mean (IQR)**			
	Time interval after radiological exam	12 (2-28)	
	Time interval after biopsy of the tumor at a nonexpert center	8 (3-36)	
	Time interval after surgery outside of an expert center	9 (2-29)	
	Time interval for the whole cohort	7.9 (1-84)	
**According to simulation by** **Sar’Connect**			
	Time interval, mean (IQR)	1.4 (0-2)	
	Difference with real-life observed results, mean (95% CI)^a^	6.476 (3.0695-9.883)	<.001

^a^Mean difference calculated with 2-tailed Student *t* test for matched data.

### Exploratory Analyses

We sought to identify the determinants of the referral time interval in real life and found no statistical correlations with the time interval in standard or Bayesian analysis (Tables S3 and S4 in Multimedia Appendix 1).

## Discussion

### Principal Findings

To our knowledge, this study is the first to assess the benefice of an app for the early management of sarcoma. Sar’Connect shortened the estimated time interval to an expert center by 7.5 months and could reduce the percentage of misorientation for patient with sarcoma.

Since the beginning of the 21st century, new technologies have contributed to improving communication and data sharing and have been developed for medical purposes. Mobile health (mHealth) was conceptualized to define the use of mobile devices in the practice of medicine and public health [27]. In 2013, 97,000 mHealth apps were released, and more than 50% of those who owned a smartphone were mHealth app users [28,29]. Moreover, this development will continue to grow because more than 90% of the population owns a mobile phone [30]. To standardize the use of mHealth apps, some countries, such as France and Belgium, have defined guidelines of good practice for health apps and smart devices, which were widely used during the COVID-19 pandemic [31-33].

In general, professional expertise and education can benefit from mHealth apps [34-36]. Before treatment occurs, it is important that health professionals be aware of the pitfalls of misdiagnosed rare tumors such as sarcomas [14]. The Sar’Connect app was developed to improve sarcoma awareness and facilitate early patient referral to expert centers, even at locations far from these centers. This app is part of the French effort to promote the early and optimal management of patients with sarcoma within the French network [1].

Prior to this study, we explored the potential benefits of Sar’Connect in a retrospective local database of patients’ files discussed before 2015 at the Strasbourg center to estimate the number of patients needed for analysis. By using the same methodology as used for this cohort, we found a difference of more than 8 months. The difference between this result and recent results may be explained by the difficulty of obtaining a precise date for the first medical description of an STT. In our study, we performed an analysis without outlier values (described as values over 1.5 times the IQR plus the third quartile or under the first quartile minus 1.5 times the IQR) to reduce this difference; 2 data points were considered extreme, and analysis excluding them did not impact the final result. The smaller potential benefits observed in this study may also be explained by geographic differences among centers and improvement in sarcoma management due to FSG awareness campaigns. Indeed, since 2010 and owing to the first French national cancer plan (2003-2009), the FSG has promoted clinical guidelines for the management of sarcoma as well as supported research [37-39]. Based on the work by Fayet et al [3], the FSG has recently emphasized the impact of the heterogeneity of referrals of patients with sarcoma to expert centers in France. In our study, patients with benign STT whose case were presented to an MTB tended to be closer to an expert center, facilitating referral. We developed Sar’Connect to improve the management of patients with sarcoma regardless of geographic disparities. By reducing diagnostic errancy and the risk of suboptimal diagnosis, treatment, and follow-up, patients living far away from expert centers should benefit the most from this mobile app [40].

Our study may be limited by population bias, as all patient data were obtained from sarcoma MTBs, and benign tumors were underrepresented. However, we primarily aimed to show that using this mHealth app may be able to reduce the estimated time interval to MTB referral for malignant tumors, which was successfully demonstrated by a reduction of more than 6 months, even when using sensitivity analyses with longer estimated time intervals. Additionally, out of 60 patients, we found a potential improvement in referral—27 (45%) patients with sarcoma immediately referred to a sarcoma MTB versus 46 (77%) patients if Sar’Connect was used. Other intrinsic characteristics will be determined by assessing the real-life use of our app.

The study was not designed to explore a potential increase in “false-positive” outcomes and the orientation of a benign tumor to a sarcoma MTB. As the app aims to avoid the misdiagnosis of malignant tumors, it was an acceptable outcome that some clinically benign STTs would be referred to an expert center according to the algorithm. Moreover, as all patients included in the analysis were ultimately referred to a sarcoma MTB and Sar’Connect only recommended immediate referral for 13 (72%) patients, we hope that the rise in suboptimal references will not be significant.

Reducing the time interval and avoiding nonoptimal initial surgeries are critical for the management of patients with sarcoma. Using real-life data from MTB databases compared to the Sar’Connect recommendations is relevant for exploring the benefits of such an mHealth app. Although our study was not designed to show any survival benefit, substantial data in the literature emphasize how critical the time interval is for the optimal management of patients with sarcoma [8,14,40-44].

mHealth app development is increasingly important, notably in oncology, and the opportunities offered by mHealth enable a wide range of issues to be addressed. For example, digital versions of patient reported outcomes (PROs), or e-PROs, are highly valuable tools in clinical research, and these data are easy to collect owing to their app. Indeed, in a lung cancer population, the use of PROs for symptoms combined with a clinically based algorithm led to an earlier diagnosis of relapse, with a median overall survival improvement of 6 months [45,46]. In palliative care, telemedicine and mHealth also improve symptom management for patients and families [47].

Other apps exist to promote the management of STT. For example, Sarculator predicts overall survival and metastatic risk in patients who undergo surgical resection because of validated nomograms [48]. Another example is Persarc, a recent mHealth app that helps experts debate STS cases through a mobile device [49]. Sar’Connect was inspired by these pioneering mHealth apps. All of these apps are already available and can easily be distributed to health professionals. Our study and those published for other apps show that a simple and user-friendly mHealth app can improve the management of patients with STS to improve the prognosis of this rare tumor.

### Conclusion

This study showed a potential benefit of more than 7 months reduction when referring patients with sarcoma to expert centers using the mobile app Sar’Connect. Our mHealth app is an example in which digital health is a useful tool to reduce disparities in the optimal management for patients with sarcoma.

## Appendix

Multimedia Appendix 1 Supplementary materials.

## Abbreviations

FSG: French Sarcoma Group
mHealth: mobile health
MRI: magnetic resonance imaging
MTB: multidisciplinary tumoral board
PRO: patient reported outcome
STS: soft tissue sarcoma
STT: soft tissue tumor

## References

[1] Honoré C, Méeus P, Stoeckle E, Bonvalot S (2015). Le sarcome des tissus mous en France en 2015: épidémiologie, classification et structuration de la prise en charge. Soft tissue sarcoma in France in 2014: epidemiology, classification and organization of clinical care. Article in French. J Chir Visc.

[2] Gatta G, Capocaccia R, Trama A, Martínez-García Carmen, RARECARE Working Group (2010). The burden of rare cancers in Europe. Adv Exp Med Biol.

[3] Fayet Y, Coindre J, Dalban C, Gouin F, de Pinieux G, Farsi F, Ducimetière Françoise, Chemin-Airiau C, Jean-Denis M, Chabaud S, Blay J, Ray-Coquard I (2018). Geographical accessibility of the referral networks in France. intermediate results from the IGéAS research program. Int J Environ Res Public Health.

[4] Gronchi A, Miah AB, Dei Tos AP, Abecassis N, Bajpai J, Bauer S, Biagini R, Bielack S, Blay JY, Bolle S, Bonvalot S, Boukovinas I, Bovee JVMG, Boye K, Brennan B, Brodowicz T, Buonadonna A, de Álava E, del Muro XG, Dufresne A, Eriksson M, Fagioli F, Fedenko A, Ferraresi V, Ferrari A, Frezza AM, Gasperoni S, Gelderblom H, Gouin F, Grignani G, Haas R, Hassan AB, Hecker-Nolting S, Hindi N, Hohenberger P, Joensuu H, Jones RL, Jungels C, Jutte P, Kager L, Kasper B, Kawai A, Kopeckova K, Krákorová D A, Le Cesne A, Le Grange F, Legius E, Leithner A, Lopez-Pousa A, Martin-Broto J, Merimsky O, Messiou C, Mir O, Montemurro M, Morland B, Morosi C, Palmerini E, Pantaleo MA, Piana R, Piperno-Neumann S, Reichardt P, Rutkowski P, Safwat AA, Sangalli C, Sbaraglia M, Scheipl S, Schöffski P, Sleijfer S, Strauss D, Strauss S, Sundby Hall K, Trama A, Unk M, van de Sande MAJ, van der Graaf WTA, van Houdt WJ, Frebourg T, Casali PG, Stacchiotti S, ESMO Guidelines Committee‚ EURACAN and GENTURIS (2021). Soft tissue and visceral sarcomas: ESMO-EURACAN-GENTURIS Clinical Practice Guidelines for diagnosis, treatment and follow-up. Ann Oncol.

[5] Kandel R, Coakley N, Werier J, Engel J, Ghert M, Verma S, Sarcoma Disease Site Group of Cancer Care Ontario’s Program in Evidence-Based Care (2013). Surgical margins and handling of soft-tissue sarcoma in extremities: a clinical practice guideline. Curr Oncol.

[6] Casali PG, Blay J, ESMO/CONTICANET/EUROBONET Consensus Panel of experts (2010). Soft tissue sarcomas: ESMO Clinical Practice Guidelines for diagnosis, treatment and follow-up. Ann Oncol.

[7] Wang J, Grignol VP, Gronchi A, Luo C, Pollock RE, Tseng WW (2018). Surgical management of retroperitoneal sarcoma and opportunities for global collaboration. Chin Clin Oncol.

[8] Traub F, Griffin AM, Wunder JS, Ferguson PC (2018). Influence of unplanned excisions on the outcomes of patients with stage III extremity soft-tissue sarcoma. Cancer.

[9] Charoenlap C, Imanishi J, Tanaka T, Slavin J, Ngan SY, Chander S, Dowsey MM, Goyal C, Choong PFM (2016). Outcomes of unplanned sarcoma excision: impact of residual disease. Cancer Med.

[10] Blay J, Honoré C, Stoeckle E, Meeus P, Jafari M, Gouin F, Anract P, Ferron G, Rochwerger A, Ropars M, Carrere S, Marchal F, Sirveaux F, Di Marco A, Le Nail LR, Guiramand J, Vaz G, Machiavello J, Marco O, Causeret S, Gimbergues P, Fiorenza F, Chaigneau L, Guillemin F, Guilloit J, Dujardin F, Spano J, Ruzic J, Michot A, Soibinet P, Bompas E, Chevreau C, Duffaud F, Rios M, Perrin C, Firmin N, Bertucci F, Le Pechoux C, Le Loarer F, Collard O, Karanian-Philippe M, Brahmi M, Dufresne A, Dupré A, Ducimetière F, Giraud A, Pérol D, Toulmonde M, Ray-Coquard I, Italiano A, Le Cesne A, Penel N, Bonvalot S, NETSARC/REPPS/RESOSFrench Sarcoma Group–Groupe d’Etude des Tumeurs Osseuses (GSF-GETO) Networks (2019). Surgery in reference centers improves survival of sarcoma patients: a nationwide study. Ann Oncol.

[11] Venkatesan M, Richards CJ, McCulloch TA, Perks AGB, Raurell A, Ashford RU, East Midlands Sarcoma Service (2012). Inadvertent surgical resection of soft tissue sarcomas. Eur J Surg Oncol.

[12] Umer HM, Umer M, Qadir I, Abbasi N, Masood N (2013). Impact of unplanned excision on prognosis of patients with extremity soft tissue sarcoma. Sarcoma.

[13] Dyrop HB, Safwat A, Vedsted P, Maretty-Kongstad K, Hansen BH, Jørgensen Peter Holmberg, Baad-Hansen T, Keller J (2016). Characteristics of 64 sarcoma patients referred to a sarcoma center after unplanned excision. J Surg Oncol.

[14] Zaidi MY, Ethun CG, Liu Y, Poultsides G, Howard JH, Mogal H, Tseng J, Votanopoulos K, Fields RC, Cardona K (2019). The impact of unplanned excisions of truncal/extremity soft tissue sarcomas: a multi-institutional propensity score analysis from the US Sarcoma Collaborative. J Surg Oncol.

[15] Potter BK, Adams SC, Pitcher JD, Temple HT (2008). Local recurrence of disease after unplanned excisions of high-grade soft tissue sarcomas. Clin Orthop Relat Res.

[16] Morinaga S, Miwa S, Yamamoto N, Hayashi K, Takeuchi A, Igarashi K, Tada K, Langit MB, Yonezawa H, Araki Y, Asano Y, Tsuchiya H (2021). Clinical characteristics of patients with undergoing unplanned excisions of malignant soft tissue tumors. J Orthop Surg (Hong Kong).

[17] Li WQ, Fu AS, Shao DF, Zhang Q, Wang MH, Wang HY, Chen Y, Zhang C, Zhu XY, Ge YL (2019). Elevated adenosine dehydrogenase (ADH) and positive tuberculin test firstly misdiagnosed as tuberculous pleural effusion finally proved as pleural mesothelial sarcoma by thoracoscopic biopsy pathology: a case report and literature review. Clin Lab.

[18] Rijal R, Mridha AR, Arava SK, Behera C (2021). Primary intimal sarcoma of the pulmonary artery misdiagnosed as pulmonary thromboembolism: a case confirmed at medicolegal autopsy. J Forensic Sci.

[19] Perisano C, Maffulli N, Colelli P, Marzetti E, Panni AS, Maccauro G (2013). Misdiagnosis of soft tissue sarcomas of the lower limb associated with deep venous thrombosis: report of two cases and review of the literature. BMC Musculoskelet Disord.

[20] Sato D, Suga H, Takushima A (2018). Liposarcoma preoperatively diagnosed as lipoma: 10-year experience at a single institution. Dermatol Surg.

[21] Kang S, Yoo HJ, Kim H, Han I (2015). Soft tissue sarcoma misdiagnosed as benign peripheral neurogenic tumor. J Orthop Sci.

[22] Johnson CN, Ha AS, Chen E, Davidson D (2018). Lipomatous soft-tissue tumors. J Am Acad Orthop Surg.

[23] Rydholm A, Berg NO (1983). Size, site and clinical incidence of lipoma. factors in the differential diagnosis of lipoma and sarcoma. Acta Orthop Scand.

[24] Wu G, Xie R, Li Y, Hou B, Morelli JN, Li X (2020). Histogram analysis with computed tomography angiography for discriminating soft tissue sarcoma from benign soft tissue tumor. Medicine (Baltimore).

[25] Fayet Y, Chevreau C, Decanter G, Dalban C, Meeus P, Carrère Sébastien, Haddag-Miliani L, Le Loarer F, Causeret S, Orbach D, Kind M, Le Nail L, Ferron G, Labrosse H, Chaigneau L, Bertucci F, Ruzic J, Le Brun Ly V, Farsi F, Bompas E, Noal S, Vozy A, Ducoulombier A, Bonnet C, Chabaud S, Ducimetière Françoise, Tlemsani C, Ropars M, Collard O, Michelin P, Gantzer J, Dubray-Longeras P, Rios M, Soibinet P, Le Cesne A, Duffaud F, Karanian M, Gouin F, Tétreau Raphaël, Honoré Charles, Coindre J, Ray-Coquard I, Bonvalot S, Blay J (2022). No geographical inequalities in survival for sarcoma patients in France: a reference networks' outcome?. Cancers (Basel).

[26] (2021). Prise en charge radiologique des tumeurs des parties molles de l’adulte. Expertise sarcome.

[27] Nannini S, Gantzer J, Tetreau R, Blay J, Kurtz J (2021). Sar'connect a mobile app to enhance soft-tissue sarcoma patients' referral to expert centers. Article in French. Bull Cancer.

[28] Adibi S (2015). Mobile Health: A Technology Road Map.

[29] Lucivero F, Jongsma KR (2018). A mobile revolution for healthcare? setting the agenda for bioethics. J Med Ethics.

[30] Mechael PN (2009). The case for mHealth in developing countries. Innovations: Technology, Governance, Globalization.

[31] Rathbone AL, Prescott J (2017). The use of mobile apps and SMS messaging as physical and mental health interventions: systematic review. J Med Internet Res.

[32] (2016). Référentiel de bonnes pratiques sur les applications et les objets connectés en santé (mobile Health ou mHealth). Haute Autorité de Santé.

[33] (2021). Validation pyramid. mHealth Belgium.

[34] Shirke MM, Shaikh SA, Harky A (2020). Implications of telemedicine in oncology during the COVID-19 pandemic. Acta Biomed.

[35] Bhatt S, Isaac R, Finkel M, Evans J, Grant L, Paul B, Weller D (2018). Mobile technology and cancer screening: lessons from rural India. J Glob Health.

[36] Coronado GD, Rivelli JS, Fuoco MJ, Vollmer WM, Petrik AF, Keast E, Barker S, Topalanchik E, Jimenez R (2018). Effect of reminding patients to complete fecal immunochemical testing: a comparative effectiveness study of automated and live approaches. J Gen Intern Med.

[37] Sly JR, Miller SJ, Jandorf L (2014). The digital divide and health disparities: a pilot study examining the use of short message service (SMS) for colonoscopy reminders. J Racial and Ethnic Health Disparities.

[38] Penel N, Bonvalot S, Minard V, Orbach D, Gouin F, Corradini N, Brahmi M, Marec-Berard P, Briand S, Gaspar N, Llacer C, Carrere S, Dufresne A, Le Cesne A, Blay J (2020). French Sarcoma Group proposals for management of sarcoma patients during COVID-19 outbreak. Article in French. Bull Cancer.

[39] Gouin F, Lacroix H (2010). Le réseau de prise en charge des sarcomes en France. Sarcomas management network in France. Article in French. Bull Cancer.

[40] Assi T, El Rassy E, Kattan J (2018). L’adhérence aux directives de la pratique clinique dans la prise en charge des sarcomes des tissus mous au Liban : leçons à apprendre de l’expérience du Groupe sarcome français. Adherence to the clinical practice guidelines in the management of soft tissue sarcomas in Lebanon: lessons from French Sarcoma Group's experience. Article in French. Bull Cancer.

[41] Pretell-Mazzini J, Barton MD, Conway SA, Temple HT (2015). Unplanned excision of soft-tissue sarcomas: current concepts for management and prognosis. J Bone Joint Surg Am.

[42] Novais EN, Demiralp B, Alderete J, Larson MC, Rose PS, Sim FH (2010). Do surgical margin and local recurrence influence survival in soft tissue sarcomas?. Clin Orthop Relat Res.

[43] Tedesco NS, Henshaw RM (2016). Unplanned resection of sarcoma. J Am Acad Orthop Surg.

[44] Morii T, Aoyagi T, Tajima T, Yoshiyama A, Ichimura S, Mochizuki K (2015). Unplanned resection of a soft tissue sarcoma: clinical characteristics and impact on oncological and functional outcomes. J Orthop Sci.

[45] Denis F, Yossi S, Septans A, Charron A, Voog E, Dupuis O, Ganem G, Pointreau Y, Letellier C (2017). Improving survival in patients treated for a lung cancer using self-evaluated symptoms reported through a web application. Am J Clin Oncol.

[46] Denis F, Viger L, Charron A, Voog E, Dupuis O, Pointreau Y, Letellier C (2014). Detection of lung cancer relapse using self-reported symptoms transmitted via an internet web-application: pilot study of the sentinel follow-up. Support Care Cancer.

[47] Worster B, Swartz K (2017). Telemedicine and palliative care: an increasing role in supportive oncology. Curr Oncol Rep.

[48] Callegaro D, Miceli R, Bonvalot S, Ferguson P, Strauss DC, Levy A, Griffin A, Hayes AJ, Stacchiotti S, Pechoux CL, Smith MJ, Fiore M, Dei Tos AP, Smith HG, Mariani L, Wunder JS, Pollock RE, Casali PG, Gronchi A (2016). Development and external validation of two nomograms to predict overall survival and occurrence of distant metastases in adults after surgical resection of localised soft-tissue sarcomas of the extremities: a retrospective analysis. Lancet Oncol.

[49] (2022). PERSARC. LUMC Oncologie Centrum.

