# Supplementary material for: Shortening the Time Interval for the Referral of Patients With Soft Tissue Sarcoma to Expert Centers Using Mobile Health: Retrospective Study
Source: JMIR Mhealth Uhealth. 2022 Nov 9;10(11):e40718. doi: 10.2196/40718 (PMC9685503; doi:10.2196/40718)
Supplement: Multimedia Appendix 1 [file mhealth_v10i11e40718_app1.docx]

## Multimedia Appendix 1

## Supplementary Materials

Figure S1.: Screenshot of the application Sar’Connect. Left picture: home screen with sidebar menu options for access to the algorithm for referring (“Orientation”); to addresses of expert centers (“Centres Référents”); Information about sarcoma, scientific group and patients’ association related to sarcoma (“Accompagner”); The profile of the user (“Mon Profil”); The credit of the application (“Crédits”)

Right picture: map that report all expert centers in France.


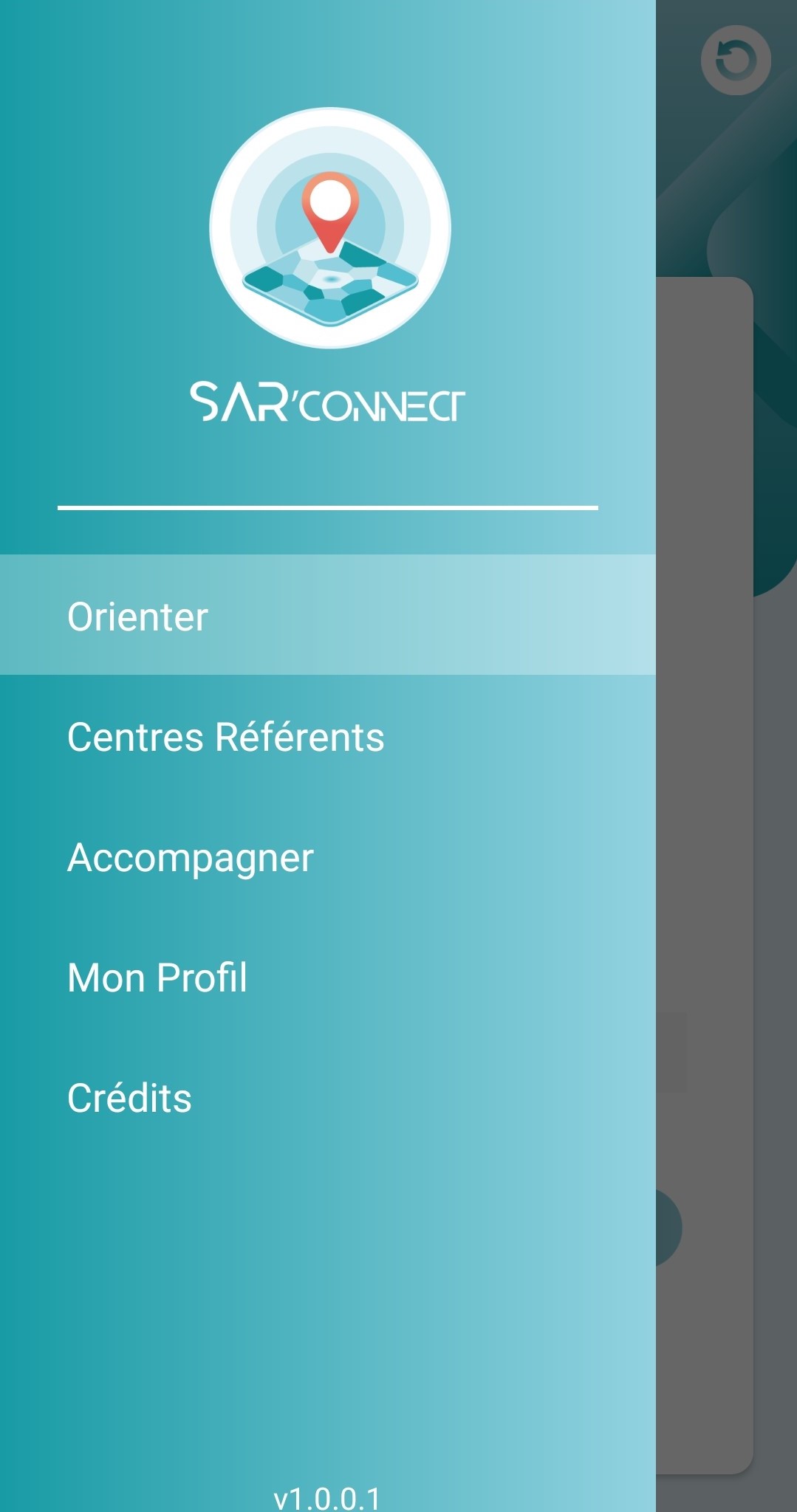

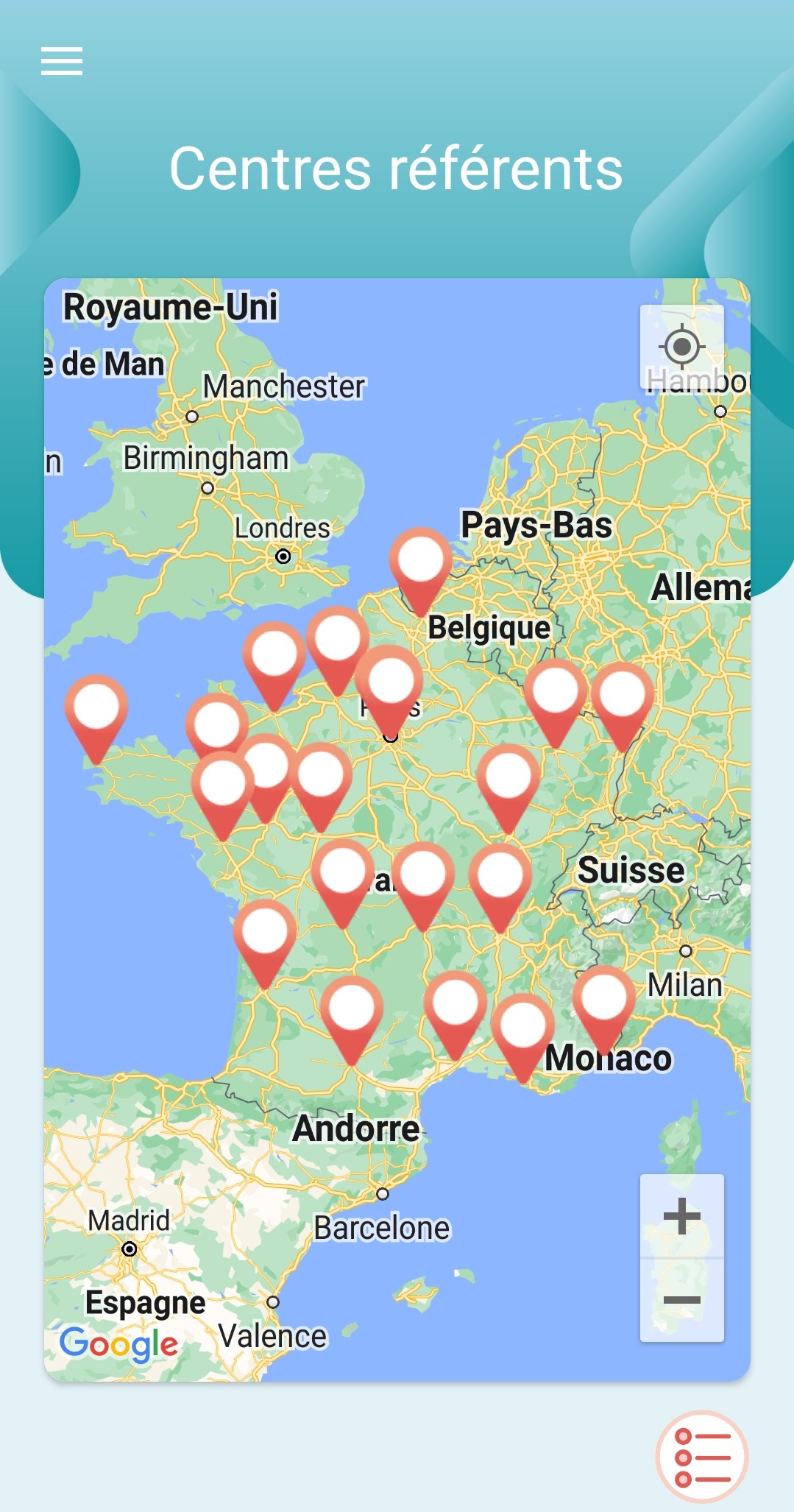


Figure S2. Summary of the main information. Abbreviation: EC = expert center; LPS = liposarcoma; LMS = leiomyosarcoma; pts = patients; NBSTM = nonbenign soft tissue tumor; PVNS = pigmented villonodular synovitis; UPS = undifferentiated pleomorphic sarcoma.


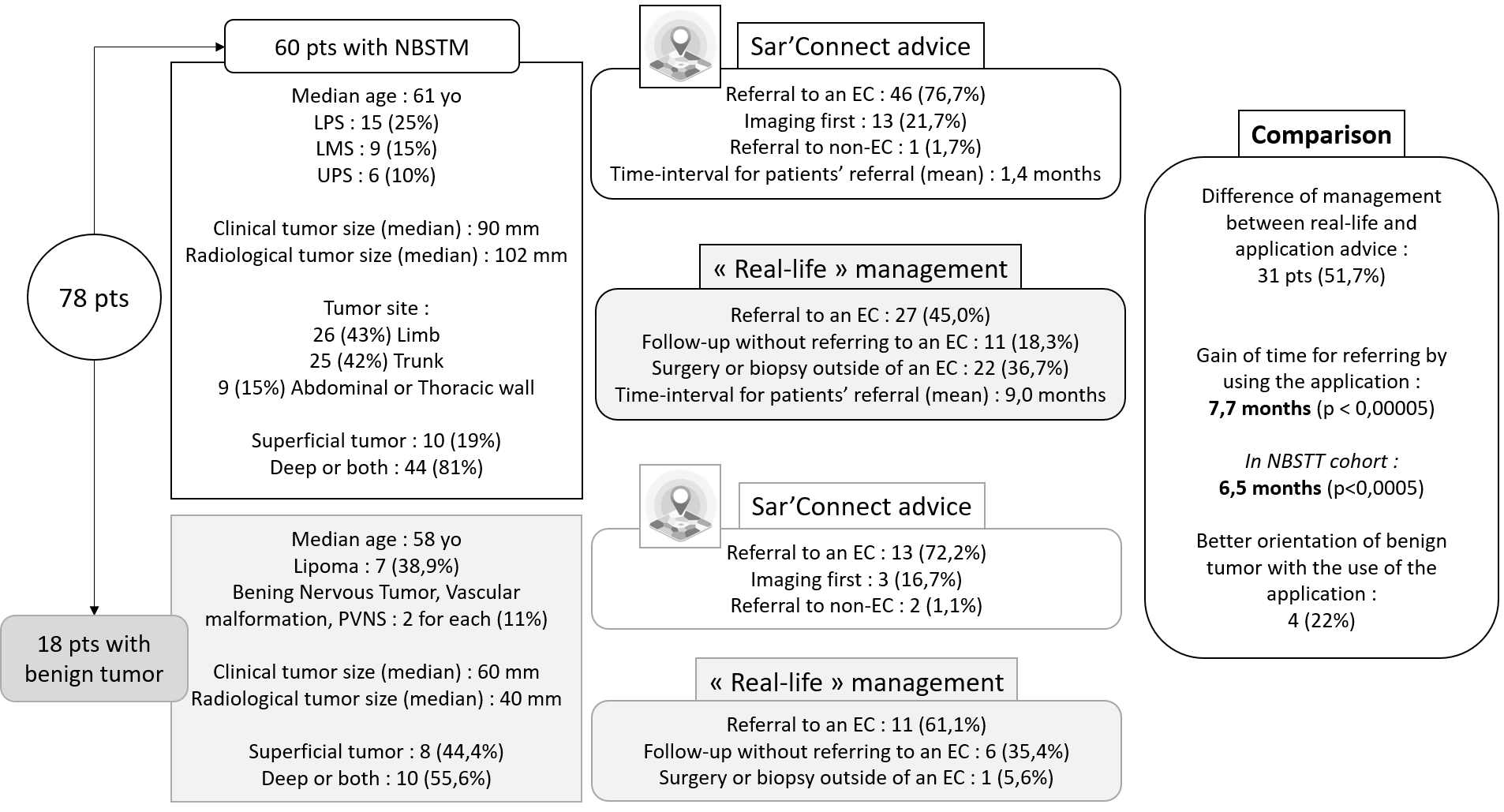


Table S1. Patient characteristics of the benign tumor cohort (N=18).

| Population characteristics | n | | % |
| --- | --- | --- | --- |
| **Median age years** (mean, range) | 58 [24;73] | | |
| < 30 years | 4 | | 22.2 |
| 30 < 60 years | 7 | | 38.9 |
| ≥ 60 years | 8 | | 44.4 |
| **Sex** |  | |  |
| Female | 9 | | 50 |
| Male | 9 | | 50 |
| **Geographical data** |  | |  |
| Distance between a patient and the nearest expert center: Median km (mean, range) | 28 [1;117] | | |
| Patient address to the nearest expert center | 18 | 100 | |
| **Histology** |  | |  |
| Lipoma | 7 | | 38.9 |
| Benign nerve tumor | 2 | | 11.1 |
| Vascular malformation | 2 | | 11.1 |
| Pigmented villonodular synovitis | 2 | | 11.1 |
| Leiomyoma | 1 | | 5.6 |
| Other***** | 4 | | 22.2 |
| **Tumor size**^§^ |  | |  |
| Clinical evaluation: Median mm (mean, range) | 60 [15;250] | | |
| Radiological evaluation: Median mm (mean, range) | 40 [10;190] | | |
| < 30 mm | 3 | | 20.0 |
| 30–50 mm | 5 | | 33.3 |
| ≥ 50 mm | 8 | | 53.3 |
| **Localization ^#^** |  | |  |
| Limb | 10 | | 55.6 |
| Trunk | 2 | | 11.1 |
| Abdominal or thoracic wall | 6 | | 33.3 |
| **Depth on radiological exam ^∆^** |  | |  |
| Superficial tumor | 8 | | 44.4 |
| Deep tumor or both superficial and deep | 10 | | 55.6 |
| **Clinical manifestation^▲^** |  | |  |
| Pain | 0 | | 0 |
| Progression of the mass | 11 | | 61.1 |
| Hardness | 6 | | 33.3 |
| Shrinkage of the mass | 1 | | 5.6 |
| Recurrence | 0 | | 0 |
|  |  | |  |

*: Other histology includes myofibroblastoma, Masson’s tumor, granulomatosis and abscess; § N = 16 (2 missing data); # Trunk localization includes the perineum, mediastinum, retroperitoneum, para-testicular and spermatic cord; **∆:** N = 15 including 3 nonpalpable soft tissue tumors (3 missing data); **▲:** patients could have more than 1 manifestation.

Table S2. Patient follow-up and referrals in the benign tumor cohort (N=18).

| Details on the first description of a soft tissue tumor (STT) | N | % |
| --- | --- | --- |
| **Radiological exam*** |  |  |
| Ultrasound echography | 12 | 66.7 |
| MRI | 14 | 77.8 |
| Computed tomography | 4 | 22.2 |
| None | 0 | 0 |
| **Results of radiological exam**^§^ |  |  |
| Atypical or suspicious aspect of STT | 11 | 61.1 |
| Homogeneous adipose or typical aspect of pseudotumor | 7 | 38.9 |
| **Medical decision after discovery of the mass** |  |  |
| Follow-up without radiological exam | 3 | 16.7 |
| Follow-up with periodic radiological exam | 3 | 16.7 |
| Biopsy in a nonexpert center | 0 | 0 |
| Surgery outside of an expert center | 1 | 5.6 |
| Referral to an expert center | 11 | 61.1 |
| **Results of Sar’Connect according to first encounter data** |  |  |
| Referral to an expert center | 13 | 72.2 |
| Performing radiological exam (MRI or echography) | 3 | 16.7 |
| Referral to a nonexpert center | 2 | 11.1 |
| **Difference between real-life decisions and Sar’Connect advice^∆^** |  |  |
| Difference   - With better orientation by the application | 9  4 | 50.0  22.2 |
| No difference | 9 | 50.0 |

*: Patients could have more than one radiological exam; § N = 18, suspicious aspect of a soft tissue tumor (STT) could contain heterogeneous tissue, anarchic vasculature, enhancement, and thick wall. Pseudotumor includes synovial or rheumatism degeneration, vascular or lymphatic malformation, elastofibroma, Morton’s neuroma, hemangioma, schwannoma, glomus tumor; **∆:** Difference of decision is based on groups: optimal decision (referral outside of an expert center) and nonoptimal one (benign soft tissue tumor patient referred to an expert center).

Table S3. Exploratory analysis for variables that might influence the time interval before referring a patient to an expert center.

| **Correlation test for explaining the time interval before referral in real life (correlation factor ρ and p value)** | |
| --- | --- |
| Distance between a patient’s address and the nearest expert center | -0.0402 (p=0.7270) |
| Age | 0.0383 (p=0.7393) |
| Number of clinical signs | -0.4610 (p=0.6461) |
| Size of the tumor | 0.1773 (p=0.1334) |
| Depth of the tumor | 0.1724 (p=0.1476) |
| Imaging before patient referral | 0.001 (p=0.9932) |

Tests were performed with a Pearson test. No variable had a significant correlation, as determined by a p value < 0.005.

Table S4. Evaluation using multiple Bayesians linear regression for predicting the time interval before referral in real life.

| **Evaluation using multiple Bayesians linear regression for predicting the time interval before referral in real life: median factor linked to the variable (credibility interval)** | |
| --- | --- |
| Distance between a patient’s address and the nearest expert center | -0.001 [-0.029;0.026] |
| Age | 0.065 [-0.107;0.235] |
| Histologic results (benign vs. nonbenign) | -4.316 [-11.970;3.097] |
| Size of the tumor > 50 mm | 0.247 [-1.635;2.795] |
| Imaging before patient referral | 0.489 [-7.992;8.847] |

Tests were performed using an MCMC method. Autocorrelation and convergence were verified. No variable had a significant impact on the time interval, as determined by a credibility interval including 0.
